# Meckel's diverticulum diagnosed by double‐balloon enteroscopy: A single‐center retrospective study in Taiwan

**DOI:** 10.1002/jgh3.12697

**Published:** 2021-12-29

**Authors:** Kai‐Chih Chang, Chia‐Hsi Chang, Jen‐Wei Chou, Yi‐Hua Wu, Po‐Ju Huang, Ken‐Sheng Cheng

**Affiliations:** ^1^ Center for Digestive Medicine, Department of Internal Medicine China Medical University Hospital Taichung Taiwan; ^2^ Division of Gastroenterology and Hepatology, Department of Internal Medicine Asia University Hospital Taichung Taiwan; ^3^ Taiwan Association for the Study of Small Intestinal Diseases (TASSID) Taoyuan Taiwan; ^4^ School of Medicine China Medical University Taichung Taiwan

**Keywords:** capsule endoscopy, double‐balloon enteroscopy, Meckel's diverticulum, Meckel's scan, obscure gastrointestinal bleeding

## Abstract

**Background and Aim:**

Meckel's diverticulum (MD) is a common congenital abnormality of the gastrointestinal (GI) tract. Although a few patients with MD present symptoms, preoperative diagnosis of MD is a clinical challenge because of its endoscopic inaccessibility. The aim of the present study was to investigate patients with MD diagnosed by double‐balloon enteroscopy (DBE) in Taiwan.

**Methods:**

We conducted a retrospective study in a tertiary referral center in middle Taiwan. The clinical characteristics, endoscopic features, histopathological findings, treatment methods, and outcomes of patients with MD diagnosed by DBE were analyzed.

**Results:**

A total of 14 male patients with MD diagnosed by DBE were enrolled. The mean age of all patients was 32.3 years. GI bleeding (78.6%) accounted for the major indication of DBE, followed by abdominal pain and Crohn's disease follow‐up. The mean distance between the ileocecal valve and MD was 68.9 cm. The average length of 12 patients with surgically resected MD was 5.2 cm. The diagnostic yields of the other modalities excepting DBE are as follows: capsule endoscopy, 50%; Meckel's scan, 11.1%; computed tomography, 16.7%; small bowel series, 0%; and angiography, 33.3%. MD presented as a large ostium in 13 patients (92.9%), a small ostium in 1 patient (7.1%), and bleeding signs in 10 patients (71.4%). Twelve patients (85.7%) underwent surgical treatment and 2 patients (14.3%) received conservative treatment. Heterotopic gastric tissue was identified in 4 patients (28.6%).

**Conclusion:**

The present study showed that DBE is a more powerful modality in detecting MD than the other conventional modalities in Taiwan.

## Graphical abstract

Meckel's diverticulum (MD) is a rare cause of small bowel bleeding in adult patients. Furthermore, it is a clinical challenge because of its endoscopic inaccessibility. Double‐balloon enteroscopy is a more powerful modality in diagnosing MD than other conventional modalities.

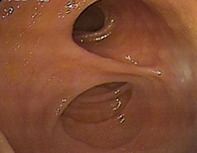

## Introduction

Meckel's diverticulum (MD) is the result of incomplete atrophy of the omphalomesenteric duct.^1^ From some previous autopsy reports, MD occurs in about 2–4% of the general population.^2^, ^3^ The majority of patients with MD are asymptomatic during their lifetime. However, 4–6% will have complication such as gastrointestinal (GI) bleeding, intestinal obstruction, intussusception, diverticulitis, enteroliths, perforation, fistula, and tumors.^4^, ^5^, ^6^ In a study reported by Mackey *et al*., they found that 16.9% of patients with MD developed symptoms.^7^ In the past, the preoperative diagnosis of MD was a challenge for most clinicians. The major reasons are due to the deep location, anatomical tortuosity, and endoscopic inaccessibility of the small intestine. Since the newly developed modality of double‐balloon enteroscopy (DBE) was introduced into the world, some patients with MD diagnosed by DBE were reported in the English literature.^8^, ^9^, ^10^ In Taiwan, DBE was first introduced in 2003. Thus, the aim of our present study was to investigate the clinical characteristics, anatomic appearances, endoscopic features, histopathological findings, treatment methods, and clinical outcomes of patients with MD diagnosed by DBE at a single medical center in middle Taiwan.

## Materials and methods

We retrospectively conducted a single‐center study for patients with MD diagnosed by DBE at China Medical University Hospital, a tertiary referral center in middle Taiwan, between July 2008 and December 2020. A total of 840 patients underwent DBE during the study period. Finally, 14 consecutive patients with MD diagnosed by DBE were enrolled in our present study. The clinical characteristics, endoscopic features, anatomical appearance, histopathological findings, treatment methods, and clinical outcomes of these patients were analyzed and discussed. All methods were carried out in accordance with relevant guidelines and regulations. Informed consent was obtained from all subjects. The methods section that the research was carried out in accordance with the Helsinki Declaration.

### Statistical analysis

The results were expressed as the mean ± SD, ranges, median, or percentages. Continuous variables were represented as the mean ± SD unless otherwise stated. Categorical variables were represented as frequency analysis, *n* (%). All statistical analyses were performed using the Statistical Package for the Social Sciences version 19.0 (SPSS Inc., Chicago, IL, USA).

## Results

Finally, a total of 14 patients with MD diagnosed by DBE were enrolled in our present study. The clinical characteristics of all our patients with MD are shown in Table 1. As for gender, all these 14 patients were male. In regard to age, the mean age of all our patients with MD was 32.3 ± 10.8 years (range, 18–54 years). We also made a subgroup analysis of different diagnostic age: Our results showed that 12 patients (85.7%, 12/14) were ≥20 years old and 2 patients (14.3%, 2/14) were <20 years old. Regarding the comorbidities, most all patients (78.6%, 11/14) had no major comorbid diseases on presentation and 3 patients (21.4%, 3/14) had comorbidities (1 had thalassemia, 1 had Crohn's disease, and 1 had idiopathic thrombocytopenic purpura). In the analysis of indication for DBE, GI bleeding (78.6%, 11/14) accounted for the majority in our all patients, followed by abdominal pain in 4 patients (28.6%, 4/14), and Crohn's disease follow‐up in 1 patient (7.1%, 1/14).

**Table 1:** Clinical characteristics of patients with MD diagnosed by DBE (*n* = 14)

Patient characteristics	No. of patients (%)
Age	
Mean age ± SD, years [range]	32.3 ± 10.8 [18–54]
≧20 years	12 (86)
<20 years	2 (14)
Sex	
Male	14 (100)
Female	0 (0)
Symptoms	
GI bleeding	11 (77)
Abdominal pain	4 (29)
No	1 (7.1)
Duration of symptom onset	
Mean time ± SD, weeks [range]	42.9 ± 69.6 [0.14–240]
Comorbidities	
Healthy	11 (78.6)
Crohn's disease	1 (7.1)
ITP	1 (7.1)
Thalassemia	1 (7.1)

DBE, double‐balloon enteroscopy; ITP, idiopathic thrombocytopenia purpura; MD, Meckel's diverticulum.

The diagnostic methods, endoscopic features, and histopathological findings of all patients with MD diagnosed by DBE are shown in Table 2. As for the diagnostic insertion directions of DBE, total 14 patients with MD were all diagnosed via a retrograde approach. In the analysis of endoscopic features, our results showed that 13 patients (92.9%, 13/14) with MD presented with a big ostium and 1 patient (7.1%, 1/14) presented with a small ostium. In the analysis of mucosal appearances in an MD, we identified that 10 patients (71.4%, 10/14) had mucosal ulcers/erosions or visible vessels in an MD, indicating the evidence of recent bleeding. We further analyzed patients with complaints of GI bleeding, 100% of these patients had mucosal ulcers/erosions or visible vessels. By contrast, we identified that 4 patients (28.6%, 4/14) had no mucosal ulcers/erosions or visible vessels in an MD. In the analysis of distribution locations, our all patients with MD were all located in the ileum. Regarding the distance between the MD and ileocecal valve, the average distance was 68.9 ± 40.2 cm (range, 35–200 cm). In the subgroup analysis, we found that the distance to be ≤60 cm in 11 patients (78.5%, 11/14) and the distance was >60 cm in 3 patients (21.4%, 3/14). The average length of 12 patients with surgically resected MD was 5.2 ± 1.9 cm (range, 3–8 cm). As for the heterotopic tissues of MD, we analyzed the findings of histopathological characters from the endoscopic biopsy specimens and surgical resection specimens, or reactivity of technetium‐99 m pertechnetate scintigraphy (so‐called Meckel's scan). As a result, we found that heterotopic gastric tissue was identified in 4 of 14 patients (28.6%).

**Table 2:** The endoscopic approach, anatomical locations, endoscopic features, and histopathologic findings of patients with Meckel's diverticulum diagnosed by DBE (*n* = 14)

Patient characteristics	No. of patients (%)
Insertion direction of DBE	
Retrograde	14 (100)
Antegrade	0 (0)
Orifice pattern of MD	
Big ostium	13 (92.9)
Small ostium	1 (7.1)
Bleeding signs of MD	
Ulcers/erosions/vessels	10 (71.4)
No	4 (28.6)
The distance between the ileocecal valve and MD^†^	
Average distance ± SD, cm [range]	68.9 ± 40.2 [35–200]
The length of MD^%^	
Mean length ± SD, cm [range]	5.2 ± 1.9 [3–8]
Heterotopic tissues in the MD^†^	
Gastric mucosa	4 (28.6)
Pancreatic mucosa	0 (0)
Colonic mucosa	0 (0)
No	10 (71.4)

DBE, double‐balloon enteroscopy; MD, Meckel's diverticulum.

^†^ All 14 measurable cases by DBE or surgical findings.

^‡^ Including findings by Meckel's scan or histopathology.

^%^ Twelve surgically resected cases.

The diagnostic yields of different modalities, treatment methods, and clinical outcomes of patients with MD diagnosed by DBE are shown in Table 3. With respect to the detection rates in other diagnostic modalities excepting DBE, abdominal computed tomography (CT) was performed in 12 of the 14 patients (85.7%), but only 2 (16.7%, 2/12) had positive findings; Meckel's scan was performed in 9 of the 14 patients (64.3%), but only 1 (11.1%, 1/9) had a positive finding; small bowel series was performed in 8 of the 14 patients (57.1%), but none of them had positive finding; capsule endoscopy (CE) was performed in 2 of the 14 patients (14.3%), but only 1 (50%, 1/2) had a positive finding; digital angiography was performed in 3 of the 14 patients (21.4%), but only 1 (33.3%, 1/3) had a positive finding.

**Table 3:** The diagnostic yields of different modalities, treatment methods and clinical outcomes of patients with MD diagnosed by DBE (*n* = 14)

Patient characteristics	No. of patients (%)
Utilized diagnostic procedures	
DBE	14 (100)
Abdominal CT	12 (85.7)
Meckel's scan	9 (64.3)
CE	2 (14.3)
Angiography	3 (21.4)
Small bowel series	8 (57.1)
Yields of diagnostic procedures	
DBE	14/14 (100)
Abdominal CT	2/12 (16.7)
Meckel's scan	1/9 (11.1)
CE	1/2 (50.0)
Angiography	1/3 (33.3)
Small bowel series	0/8 (0)
Treatment methods	
Surgical treatment	12 (85.7)
Conservative treatment	2 (14.3)

CE, capsule endoscopy; CT, computed tomography; DBE, double‐balloon enteroscopy; MD, Meckel's diverticulum.

In the analysis of treatment methods, we found that 12 patients (85.7%, 12/14) with MD underwent the surgical resection of MD after endoscopic diagnosis. In the surgical treatment group, a laparoscopy was performed in 9 patients (75%, 9/12), a laparotomy was performed in 2 patients (16.7%, 2/12), and a laparoscopy which converted to laparotomy was performed in 1 patient (8.3%, 1/12). In contrast, 2 patients (14.3%, 2/14) declined surgical treatment and chose conservative management. In the analysis of clinical outcomes, no instances of postoperative complication or recurrence of symptoms were noted in the surgical treatment group, however, a patient with MD had recurrent GI bleeding 10 months later after discharge in the conservative treatment group.

## Discussion

MD is a relatively common congenital malformation of the GI tract. The prevalence of MD is usually equal distribution in both sexes, but it has a male predominance in symptomatic patients with a male to female ratio ranging from 2:1 to 5:1.^11^, ^12^ In our present study, we found that our patients with MD were all males and most were symptomatic. Among complications of MD, GI bleeding occurs predominantly in children, while inflammation and obstruction occur in adults.^3^, ^4^, ^5^, ^7^ When patients present with obscure GI bleeding accompanied by MD, it is usually difficult to determine whether MD is the cause of bleeding or not because most patient with MD are asymptomatic. Therefore, we need more additional information of an MD based on the endoscopic observations and features.

The preoperative diagnosis of MD was a clinical challenge for most clinicians in the past. Conventionally, diagnostic modalities for an MD include a small bowel series, Meckel's scan, abdominal CT, angiography, and even surgery. On a small bowel series, MD may manifest as a blind‐ending pouch or a polypoid filling defect arising from the anti‐mesenteric side of the ileum.^13^ However, MD can be misdiagnosed via this examination because of its small ostium, its filling with intestinal contents or rapid peristalsis of the small intestine. Moreover, the detection of ulcerations in an MD using a small bowel series is usually impossible. In our present study, we performed a small bowel series in 57.1% of all patients; however, none of them was diagnosed as MD via this method. On abdominal CT, MD may be shown as a blind‐ending fluid or gas‐filled structure in continuity with the small intestine, but it is also difficult to distinguish from the normal small intestine in uncomplicated cases.^14^ Our results showed that abdominal CT was the most commonly utilized diagnostic modality in patients with MD (85.7%); however, its diagnostic yield was only 16.7%. On digital angiography, it may demonstrate a persistent vitellointestinal artery in most patients with MD who present with chronic GI bleeding.^15^ Moreover, this procedure can be useful for applying embolization treatment to an overt bleeding vessel. In our present study, we performed a digital angiography in 21.4% of all patients, and it had a diagnostic yield of 33.3%. Meckel's scan is a useful modality for detecting the existence of MD because of its reactivity with the gastric mucosa. Although Meckel's scan showed a high sensitivity rate (85–90%) in pediatric patients, it had a low sensitivity rate (< 60%) in adult patients.^16^ In our present study, we performed Meckel's scan in 64.3% of all patients with MD, and it had a diagnostic yield of just 11.1%, which is in line with the previous reports in adult patients. On endoscopic examination, conventional push‐type enteroscopy or a colonoscopy is usually difficult to identify an MD because they cannot reach the ileum. Since the newly developed diagnostic modalities, including DBE and CE, were introduced to the world in the past two decades, the diagnosis of small bowel disorders has evolved markedly. In the past, a diagnosis of MD by DBE or CE was usually case reports and lacked a large case series in the English literature. There are two major reasons for explaining this situation. First, symptomatic MD is relatively rare in adult patients. Second, DBE and CE are rarely performed in children with symptomatic MD because pediatricians have less enteroscopic experiences in clinical practice. Therefore, an MD diagnosed by DBE or CE is usually a chance finding. Although CE is a noninvasive modality used to examine the entire small intestine, the diagnostic yield of CE for an MD was limited until now. Mylonaki *et al*. first reported an MD detected by CE and described it as a “black hole” or having a “blood‐filled” appearance.^17^ Furthermore, it is also difficult to detect an ulcer in an MD via CE because of its rapid peristalsis. Montemaggi *et al*. have shown a circular ulcer in an MD detected by CE.^18^ Although CE can detect an ulcer or not in an MD, it lacks the capacity of taking tissue samples and could even become trapped in the MD.^19^ Despite the fact that only a few patients (14.3%) underwent CE in our present study, the diagnostic yield of CE was up to 50%, which was the highest diagnostic yield of all modalities excepting DBE. In contrast to CE, DBE can not only detect an MD, but also has the capacities of tissue sampling and endoscopic treatment. Yamamoto *et al*. first reported a case of MD diagnosed by DBE in 2001.^8^ Later, several authors have published some small case series of MD diagnosed by DBE in the English literature.^20^, ^21^, ^22^ In comparison with these previous small case series reports, He *et al*. reported a large case series of 64 patients with MD diagnosed by DBE before surgery.^23^ Herein, we reported a case series of 14 patients with MD diagnosed by DBE in middle Taiwan.

**Figure 1:**
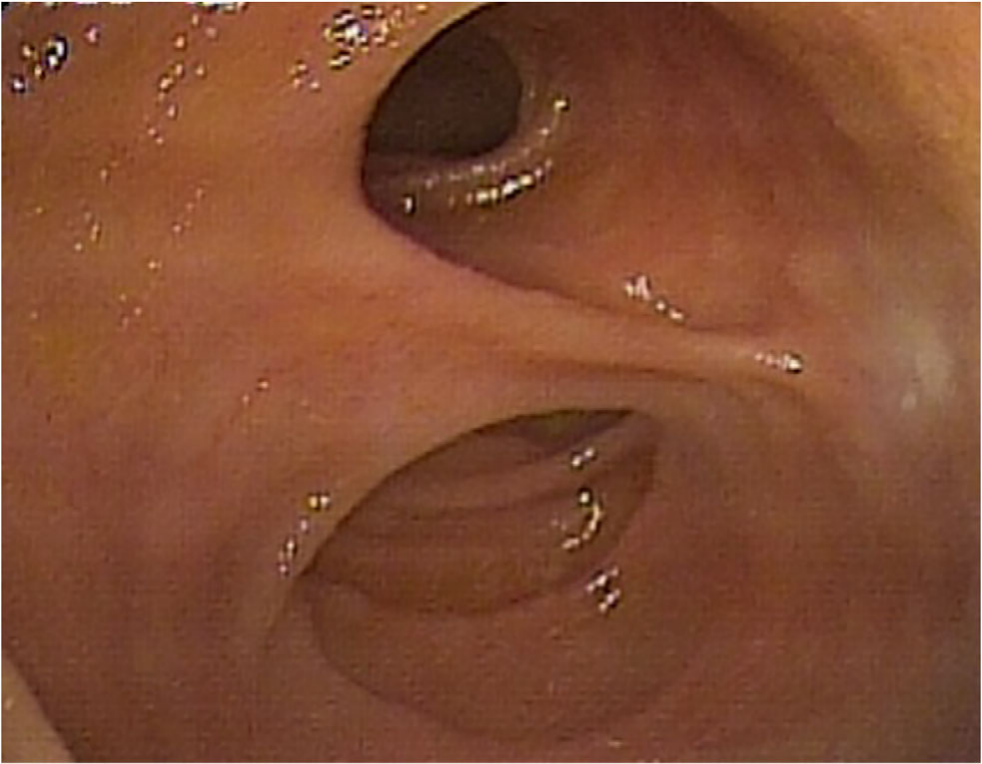
Endoscopy showing a large ostium of Meckel's diverticulum (MD) during double‐balloon enteroscopy.

**Figure 2:**
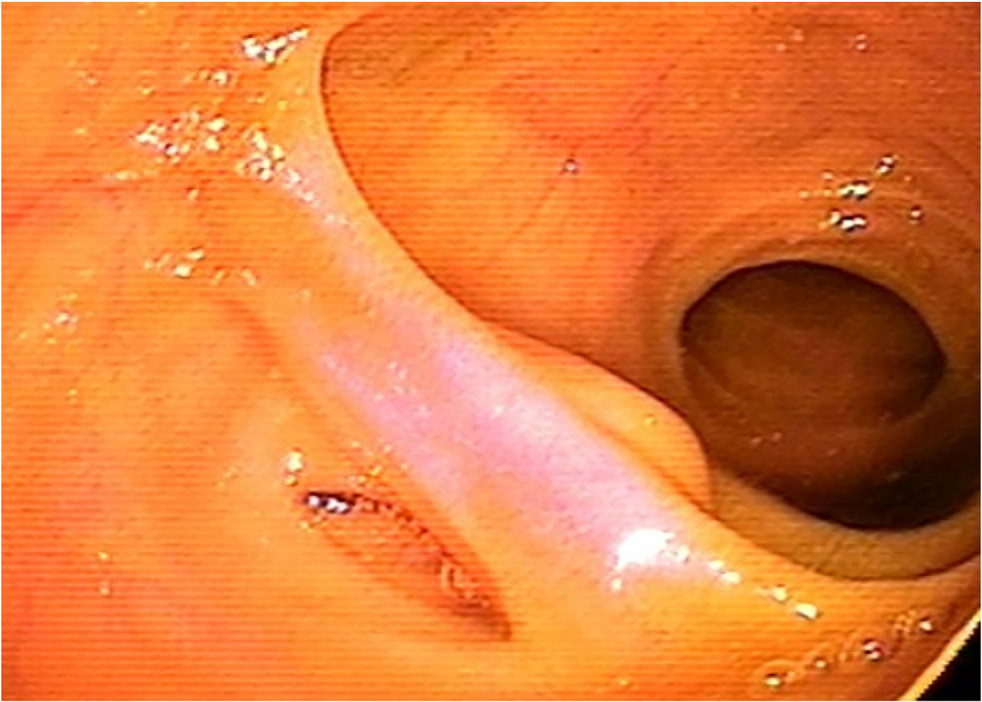
Endoscopy showing a small ostium of Meckel's diverticulum (MD) during double‐balloon enteroscopy.

**Figure 3:**
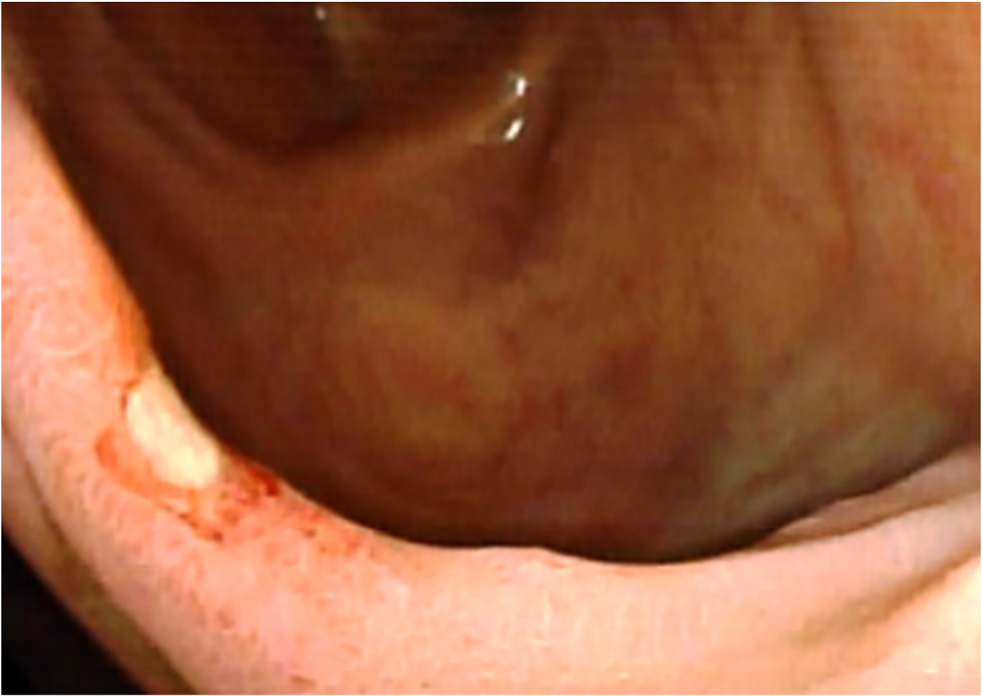
Endoscopic showing an ulcer in the margin of Meckel's diverticulum (MD) during double‐balloon enteroscopy.

Regarding the endoscopic features, MD usually presents with a large ostium but it can also present with a small ostium or an inverted polypoid mass.^8^, ^10^, ^24^ Our results showed that 92.9% of all patients with MD presented as a large ostium (Fig. 1) and only 1 patient (7.1%) presented as a small ostium (Fig. 2). Shinozaki *et al*. suggested that the detection of ulcers could be reliable evidence of GI bleeding from an MD.^21^ However, the mechanism of ulcer formation in an MD is still unclear in spite of several hypotheses. In the English literature, several authors speculated that gastric acid secreted from the heterotopic gastric mucosa may induce the ulcerations.^3^, ^25^ Moreover, *Helicobacter pylori* infection was also identified and considered as a cause of ulcerations in an MD with heterotopic gastric mucosa.^26^ However, the above two speculations are currently debated by other authors in the English literature.^27^ Manner *et al*. described another possible explanation that high mechanical irritation in the area of the tissue bridge between the ileal lumen and MD might have led to the ulceration.^20^ If MD with ulceration is detected by DBE, total enteroscopy for evaluating the entire small intestine may not be necessary. On the contrary, other bleeding sources should be investigated when no ulcers are identified in an MD. Patients with MD who do not experience recurrent bleeding after surgery could support this theory. In a pediatric study reported by Rutherford *et al*., they found that 81% of patients with complaints of GI bleeding had ulcers in the resected MD.^28^ Our results showed that 71.4% of all patients and 100% of patients with complaints of GI bleeding had bleeding signs in the MD during DBE (Fig. 3).

MD may harbor heterotopic tissues within its mucosa, including gastric, duodenal, colonic, pancreatic, and hepatobiliary tissues.^2^ Among these heterotopic tissues, heterotopic gastric tissue accounts for the most common type in symptomatic MD (45–80%).^29^ In a study reported by Yamaguchi *et al*., they found that heterotopic gastric mucosa was only identified in 30% of all patients with MD and 62% of symptomatic MD.^5^ In the present study, we found that heterotopic gastric mucosa was only identified in 28.6% of all patients by the findings of Meckel's scan, surgically resected specimens or endoscopic biopsy specimens. However, there was no heterotopic pancreatic tissue nor heterotopic colonic tissue identified in our present study.

Surgical resection is the mainstay treatment for patients with complicated MD.^30^, ^31^ Laparoscopy or laparoscopy‐assisted approach method has become a safe and effective method for treating complicated MD compared with the traditional open approach.^32^ In our present study, we found that 85.7% of all our patients with MD underwent surgical treatment, including laparoscopy (75%), laparotomy (16.7%), and laparoscopy converter to laparotomy (8.3%). No postoperative complications or recurrence of symptoms were noted in the surgical treatment group. However, surgical treatment for asymptomatic and incidentally discovered MD is still controversial until now. In a study reported by McKay, he suggested that surgical treatment of asymptomatic MD should be considered in patients younger than 50 years of age, whereas patients older than 50 years of age will be less likely to benefit from this prophylactic resection.^33^ Furthermore, Park *et al*. conducted a large study of patients with MD during surgery.^34^ They also suggested that a surgical treatment was indicated in male patients younger than 50 years of age. Conservative treatment for complicated MD was rarely reported in the English literature. Our results showed that 14.3% of all patients with MD received conservative treatment because they refused the surgical treatment. One patient with MD had recurrent GI bleeding 10 months later after diagnosis; the other one with incidental finding of MD had no any complications after the treatment of Crohn's disease.

## Conclusions

MD is an important differential diagnosis in adult patients presenting with GI bleeding, abdominal pain, fever, or other symptoms. Preoperative diagnosis of MD remains a challenge in clinical practice. According to the clinical findings in our present study, DBE is a powerful modality in detecting an MD compared to other conventional modalities. Ulcer detection in an MD by DBE is an important evidence of GI bleeding of MD. Moreover, the following minimally invasive laparoscopic resection after endoscopic diagnosis could be performed for the treatment of MD. Finally, this study is associated with some limitations. First, the main limitation in our present study is the small number of patients. Second, it is a retrospective study and some patients' records may be incomplete. Therefore, multicenter studies are needed to fully assess the efficacy and safety of DBE in the diagnosis of MD in the future.
